# Cyclooxygenase-2 immunoexpression in intestinal epithelium and lamina propria of cats with inflammatory bowel disease and low grade alimentary lymphoma

**DOI:** 10.1186/s12917-018-1486-0

**Published:** 2018-05-15

**Authors:** Jorge Castro-López, Antonio Ramis, Marta Planellas, Mariana Teles, Josep Pastor

**Affiliations:** 1 Departament de Medicina i Cirurgia Animals, Universitat Autònoma de Barcelona, 08193 Barcelona, Spain; 2 Fundació Hospital Clínic Veterinari de la Universitat Autònoma de Barcelona, 08193 Barcelona, Spain; 3 Servei de Diagnòstic de Patologia Veterinària, Departament de Sanitat i d’Anatomia Animals, Universitat Autònoma de Barcelona, 08193 Barcelona, Spain; 4 Department de Biologia Cel·lular, Fisiologia i d’Immunologia, Universitat Autònoma de Barcelona, 08193 Barcelona, Spain

**Keywords:** Feline, Chronic enteropathy, Inflammatory bowel disease, Alimentary lymphoma, Cyclooxigenase 2, COX-2

## Abstract

**Background:**

Cyclooxygenase 2 (COX-2) is an inducible isoform by cellular activation, proinflammatory cytokines and growth factors. The aims of the current study were to evaluate COX-2 immunoexpression in epithelial and lamina propria (LP) of cats with inflammatory bowel disease (IBD) and low grade alimentary lymphoma (LGAL), as well as to correlate them with clinical signs and histopathological scoring. Cats diagnosed with IBD and LGAL (2007–2013) were included in the current study. Feline chronic enteropathy activity index (FCEAI) was calculated for all cases. Control group was composed by 3 healthy indoor cats and 5 sick cats died or were euthanized (non-gastrointestinal illness). Diagnosis and classification of IBD and LGAL was established according to the WSAVA gastrointestinal standardization group template and the National Cancer Institute formulation, respectively. Furthermore, a modified WSAVA template was applied for LGAL evaluation. Immunolabelling for COX-2 (polyclonal rabbit anti-murine antibody) was performed on biopsy samples. Epithelial and LP (inflammatory or neoplastic cells) COX-2 immunolabelling was calculated according to the grade and intensity. The most representative segment scored by the WSAVA and the modified WSAVA were used for statistical analysis.

**Results:**

Significant difference was found regarding COX-2 intensity overexpression in the epithelial cells of IBD and LGAL groups when compared to control cats, but not between the groups of sick cats, whereas no differences were found regarding the grade of immunoreactivity between groups. No difference was found for COX-2 immunoexpression at the LP between all groups. However, 3 cats from LGAL group showed COX-2 expression in neoplastic cells at the LP. There were no correlations between epithelial or LP COX-2 expression and FCEAI and histological alterations.

**Conclusions:**

Increased COX-2 intensity at the epithelial cells observed in cats with IBD and LGAL may be secondary to the inflammatory response or a protective function in the intestinal reparation. COX-2 expression at the LP was presented in 33% of LGAL. This result provides a reason for further investigation concerning the role of COX-2 expression in feline alimentary lymphoma.

## Background

Inflammatory bowel disease (IBD) and low grade alimentary lymphoma (LGAL) are common causes of chronic enteropathies (CEs) in cats [1–6]. IBD is a chronic immune-mediated disease whose cause remains unknown but is likely multifactorial [1–3, 6]. Currently, alimentary lymphoma (AL) is the most common anatomic form of lymphoma and its cause is also unknown [5, 7–12]. IBD and LGAL can affect any segments of the gastrointestinal (GI) tract and clinical differentiation between them may be a challenge. Therefore histopathological diagnosis is always needed though overlapping may also occur, complicating the definitive diagnosis [3, 5, 13–16]. In addition, evolution from chronic intestinal inflammation to AL has been proposed in cats but definitive proof is lacking [9, 17].

Cyclooxygenase 2 (COX-2) is an inducible inflammatory regulator isoform by cellular activation, proinflammatory cytokines, growth factors, tumour promoters and prostaglandin mediator [18–21]. Prostaglandin E_2_, a COX-2 metabolite, has many biological roles including mediating pain, modulation of cytokine production, induction of regulators of angiogenesis, production of proinflammatory mediators and promotes tumourigenesis [22, 23]. Furthermore, overexpression of COX-2 may be a consequence of inflammation leading to increased levels of Bcl-2 and resistance to apoptosis of the cells, thus enhancing the risk of cancer [24, 25]. To the author’s knowledge, there is only one available study in cats that included 6 cases of intestinal lymphoma and described negative COX-2 immunoexpression [26], and there is no study describing COX-2 immunoexpression in feline IBD and LGAL.

The aim of the present study was to evaluate COX-2 immunoexpression at the epithelium and lamina propria (LP) of cats with IBD and LGAL. The second objective was to correlate the COX-2 immunolabelling with clinical signs and histopathological scoring.

## Methods

### Study population

Control group was composed of 3 healthy control indoor female cats (HCC, median age = 2 years; range = 1–5 years) owned by the personal staff were submitted to endoscopy prior to ovariohysterectomy and duodenal biopsies were obtained, and 5 sick cats (SC, median age = 7 years; range = 1–18 years) who died or were euthanized for unrelated GI diseases and full thickness biopsies (FTB) from duodenum, jejunum and ileum were obtained within 1 h. Cats had not received glucocorticoids (GC), chemotherapy, non-steroidal anti-inflammatory drugs (NSAIDs) or antibiotics with immunomodulatory action such as doxycycline and azithromycin previously. All these cats were recruited from the Fundació Hospital Clínic Veterinari of the Universitat Autònoma de Barcelona.

Approval consent was signed and accepted by the owners and procedures were approved by the Ethical Committee from the Faculty of Veterinary Medicine and Bioscience Engineering of Universitat Autònoma de Barcelona (CEAAH 2354).

IBD and LGAL cases of the study were collected between 2007 and 2013 from the Fundació Hospital Clínic Veterinari of the Universitat Autònoma de Barcelona. The inclusion criteria was the presence of chronic GI signs (> 3 weeks duration), complete medical history and no previous GC, chemotherapy, NSAIDs or antibiotics with immunomodulatory action treatments 6 months before the presentation. Information obtained from all cats included signalment (age, breed, sex, body weight), history, physical examination, clinicopathological testing (complete blood count, biochemistry profile and total T4 and abdominal ultrasonography). All patients were negative to feline leukaemia virus antigen and immunodeficiency virus antibodies. Cats with mild to moderate clinical signs were treated at the beginning with antiparasitic for 5 days, followed by elimination diet (novel protein or hydrolysed elimination diets) for at least 14 days to rule out parasitism and food response enteropathy, respectively. Posteriorly, endoscopy or FTB were obtained. Otherwise, severely compromised patients were submitted to intestinal biopsy after blood works and ultrasonography. These patients did not receive antiparasitics or placed on diet trials at presentation, but did during treatment in cats with IBD. Biopsies were obtained by laparotomy (duodenum, jejunum and/or ileum) or endoscopy (duodenum). Stomach and colonic biopsies were not considered in this study. Cats with extra-GI diseases were excluded from the study.

### Chronic enteropathy activity index

The feline chronic enteropathy activity index (FCEAI) was applied to all studied cats [2]. This index gave a scoring to GI signs (vomiting, diarrhoea, anorexia, weight loss, lethargy; 0 to 3 points for each sign according to severity), hyperproteinaemia (yes = 1 point, no = 0 point), hypophosphataemia (yes = 1 point, no = 0 point), increased serum alanine aminotransferase (ALT) and/or alkaline phosphatase (ALP) activities (yes = 1 point, no = 0 point). Endoscopic lesions parameter was not included because FTB were performed in most of the cats and endoscopy was not repeated. A questionnaire was filled by the owners at the first visit or phone calls. A composite score was subsequently calculated yielding values for mild (2 to 5), moderate (6 to 11) and severe (12 or greater) CE [27].

### Histopathological classification

Biopsy samples were fixed in neutral-buffered formalin and embedded in paraffin wax. Tissue was sectioned (3 μm) and stained with haematoxylin and eosin. Single board-certified pathologist (AR) reviewed all sections and was blinded to the clinical information. Previously published diagnostic algorithm was used to differentiate IBD from LGAL [16].

Biopsies from the control and IBD groups were evaluated according to the world small animal veterinary association (WSAVA) GI Standardization Group template [28]. This template only assesses the duodenal morphological features (villous stunting, epithelial injury, crypt distension, lacteal dilation and mucosal fibrosis) and inflammation changes (intraepithelial lymphocytes, LP lymphocytes and plasma cells, eosinophils, neutrophils, other cells) from the duodenum. They were scored as absent = 0, mild = 1, moderate = 2, or severe = 3. Finally, histologic severity scores were recorded and determined to be normal (score 0), mild (1–6), moderate (7–13), severe (14–20), and very severe (> 20) [29]. Jejunal and ileal biopsies were scored according to the WSAVA template as Casamian-Sorrosal and colleagues described in these segments [30].

Modified WSAVA (MWSAVA) score was used for LGAL cases that included morphological features (villous stunting, epithelial injury and crypt distension) and applied to duodenum, jejunum and ileum [31]. These features were scored as absent = 0, mild = 1, moderate = 2, or severe = 3. Total scores were classified as normal (score = 0), mild (1–3), moderate (4–6), severe (7–9), and very severe (> 10) according to a calculated proportion of the classification mentioned above.

LGAL cases were classified according to the National Cancer Institute working formulation. The number of mitoses between 0 and 5 at high-power field and small nuclear size (< 1.5X the size of a red blood cell) correspond to LGAL [32]. Furthermore, CD3 and CD20 immunophenotyping was performed in LGAL and severe IBD cases as previously described [16].

For statistical evaluation, the small intestinal segment with the higher or modified histological score of each individual was considered.

### COX-2 immunohistochemistry

**Fig. 1:**
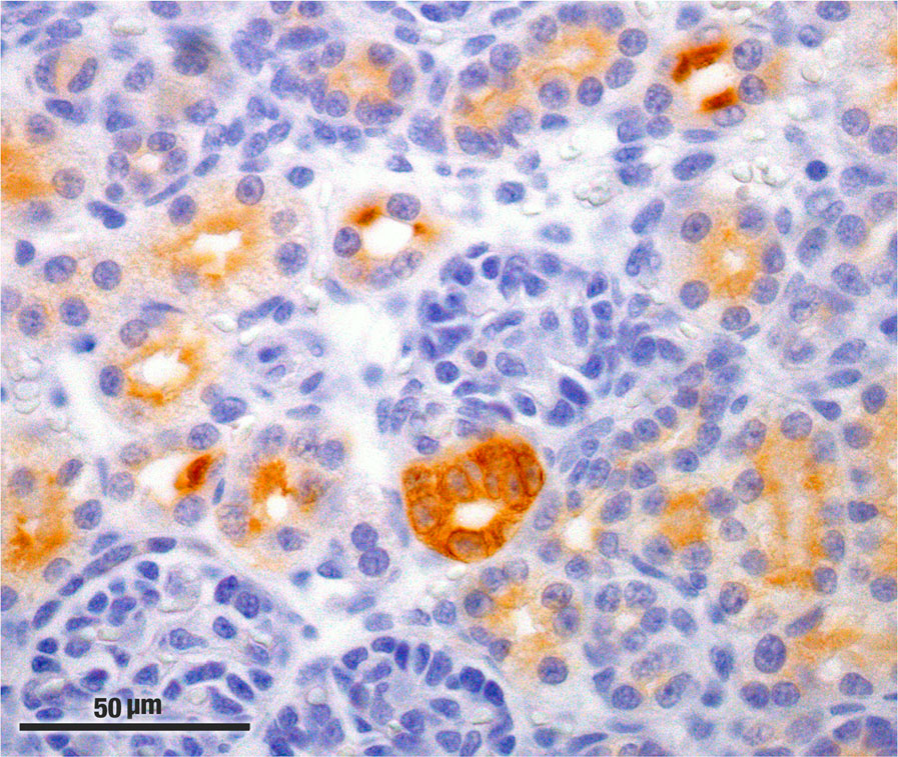
Macula densa from a foetal kidney showing marked intensity of COX-2 immunoexpression and apical border of renal tubular cells expressing moderate intensity

Sections (3 μm) were routinely deparaffinised, rehydrated and antigen retrieval at pH 6 was performed by PT-Link Automatic System (Dako Glostup, Denmark). Immunostaining was performed on a Dako Autostainer Plus, using procedures, buffers and solutions provided by the manufacturer. Primary antibody binding was detected with a standard two-layer indirect method (EnVision; DakoCytomation). Chromogen staining was developed with diaminobenzidine. Slides were counterstained with haematoxylin. The primary antibody (polyclonal rabbit anti-murine COX-2; Cayman Chemical, Ann Arbor, Michigan, USA) at a 1 in 500 dilution was used. A rabbit polyclonal antibody against *Leishmania infantum*, kindly provided by Instituto de Salud Carlos III (Madrid, Spain), was used for negative control purposes (1:3000). Sections of feline foetal kidney (Fig. 1) and cutaneous squamous cell carcinoma were used as positive controls [33–35]. COX-2 immunohistochemical staining was performed on a normal feline lymph node as a negative control.

Epithelial, inflammatory and/or neoplastic cells COX-2 immunolabelling was evaluated by a semi-quantitative assessment which included staining grade (percentage of positive cells) and intensity. Five 10X fields from each slide were evaluated. The grade (percentage) was evaluated by the following scoring system: 0 = negative; 1 = < 10% of cells staining positive; 2 = 10–30%; 3 = 31–60%; 4= > 60%. Intensity was evaluated by the following scoring system: 0 = negative; 1 = weak staining; 2 = moderately intense staining; and 3 = marked intense staining. Intensity of positive control cells was considered marked staining [26]. The final expression score was calculated multiplying the intensity with percentage and classified as weak (1–2), moderate (3–5), marked (6–8) and very marked (> 9).

### Statistical analysis

Statistical analysis was performed using SPSS statistics software (SPSS 17.0 version, Chicago, IL, USA) adopting a level of significance of *p* < 0.05. Shapiro-Wilk test were used for tested normality of the data. Non-parametric tests were applied for data that did not present a normal distribution, and median and range were used for summary. The Kruskal-Wallis test was used to compare continuous variables (FCEAI, WSAVA and MWSAVA scores, epithelial and LP COX-2 expression) between groups. The Mann-Whitney test was used as post-test analysis for the evaluation of the variation between the different groups.

## Results

A total of 28 cats met the inclusion criteria but 8 cats were eliminated because biopsy samples were unavailable. Therefore, 11 cats with IBD and 9 cats with LGAL were studied. The median age was 5 years (range = 2–12) for IBD group and 12 years (range = 8–15) for the LGAL group. LGAL group presented a slightly higher body weight (median = 4.2 kg; range = 3.00–6.26) than IBD group (median = 3.88 kg; range = 2.00–6.00). All cats were neutered, except 1 intact female and 1 intact male from the IBD group. There were 5 (45%) female and 6 (55%) male cats in the IBD group and 1 (11%) female and 8 (89%) male cats in the LGAL group. Breeds represented in the IBD group were Domestic Shorthair (DSH, 4), Domestic Longhair (3), Siamese (2), Persian (1) and Norwegian Forest (1) cats. All cats belonging to the LGAL group were DSH cats.

Endoscopy biopsies were obtained from 3 cats and FTB from 8 cats of the IBD group. Samples were obtained mostly from the duodenum (9 cats). Regarding the inflammatory cells infiltration at the LP, 8 cases had lymphoplasmacytic (73%) and 3 eosinophilic (27%) inflammation. FTB were collected in all cats with LGAL except for 1 patient. All LGAL animals were T cell lymphoma and it was most commonly diagnosed in the jejunum (6 cats out of 9), followed by duodenum (2) and ileum (1).

Median of FCEAI score obtained by LGAL group was 11 (range = 5–14) and IBD group was 9 (range = 4–12) corresponding to moderate CE, but no statistical significant difference was found (*p* = 1.000) (Table 1).

**Table 1:** FCEAI, modified and total WSAVA scores and COX-2 immunoexpression of Control, IBD and LGAL group

Group	FCEAI	Modified WSAVA score	Total WSAVA score	Intensity Epithelium	% Epithelium	Total Epithelium	Intensity LP	% LP	Total LP
Control (median)	–	0^a^	1^a^	2^a^	4^a^	8^a^	0^a^	0^a^	0^a^
IBD (median)	9^a^	1^a^	5^b^	3^b^	4^a^	12^a^	0^a^	0^a^	0^a^
LGAL (median)	11^a^	2^b^	–	3^b^	4^a^	12^a^	0^a^	0^a^	0^a^

*FCEAI* feline chronic enteropathy activity index, *WSAVA* world small animal veterinary association; *%* percentage, *LP* lamina propria, *IBD* inflammatory bowel disease, *LGAL* low grade alimentary lymphoma, *−* non score. Different letters show a significant difference (*p* < 0.05)

**Fig. 2:**
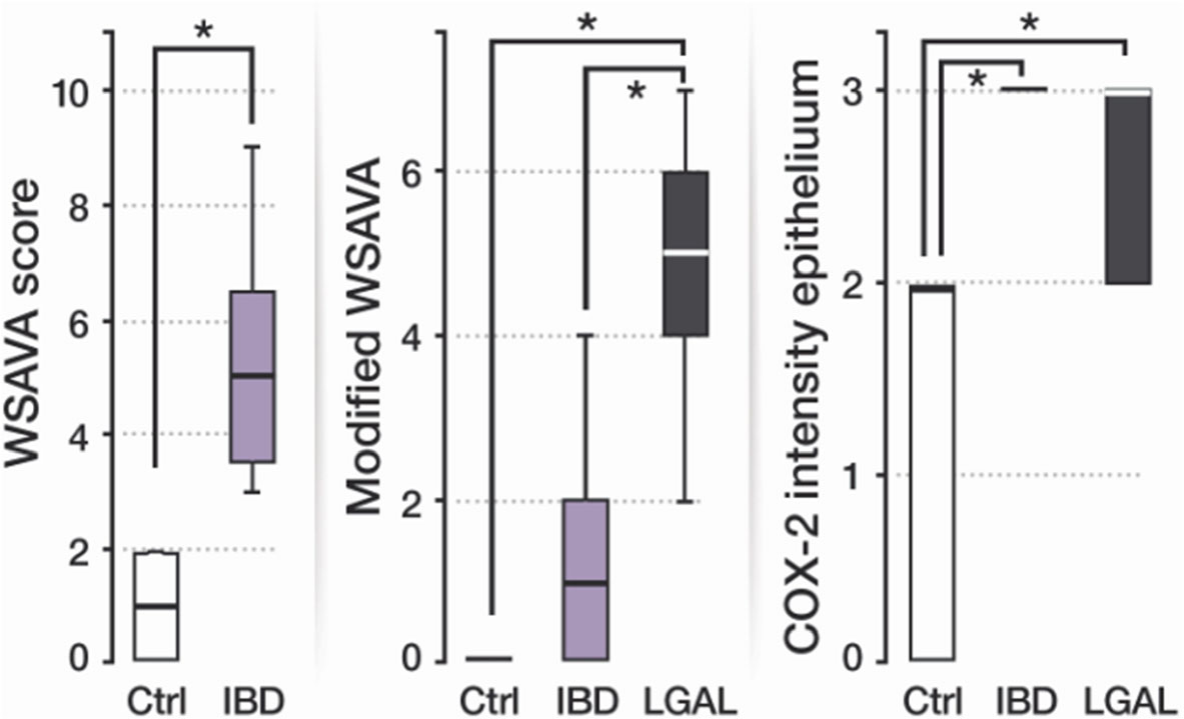
(left) WSAVA scores comparison between Control (Ctrl) and IBD group (*: significant difference (*p* < 0.05) between Control and IBD group), (center) modified WSAVA scores comparison between Control, IBD and LGAL group (*: significant difference (*p* < 0.05) between Control and LGAL group; between IBD and LGAL group) and (right) COX-2 intensity in the epithelium (*: significant difference (*p* < 0.05) between Control and IBD group; between Control and LGAL group). Box plots represent median, 25th percentil, 75th percentil, maximum and minimum. WS AVA : world small animal veterinary association; IBD: inflammatory bowel disease; LGAL: low grade alimentary lymphoma; COX-2: cyclooxygenase 2

According to the WSAVA template, IBD group showed a significant statistically higher score of morphological and inflammatory changes compared to the control group (*p* = 0.011, Table 1 and Fig. 2). Considering the MWSAVA score, that only includes the morphological features of the WSAVA template, LGAL group presented a significantly higher value than the IBD (*p* = 0.011) and control group (*p* < 0.001, Table 1 and Fig. 2). No significant difference was found between IBD and control group according to the MWSAVA score (*p* = 0.156, Table 1 and Fig. 2). No lineal correlation was found between FCEAI and total WSAVA, and MWSAVA scores (*p* > 0.05).

**Fig. 3:**
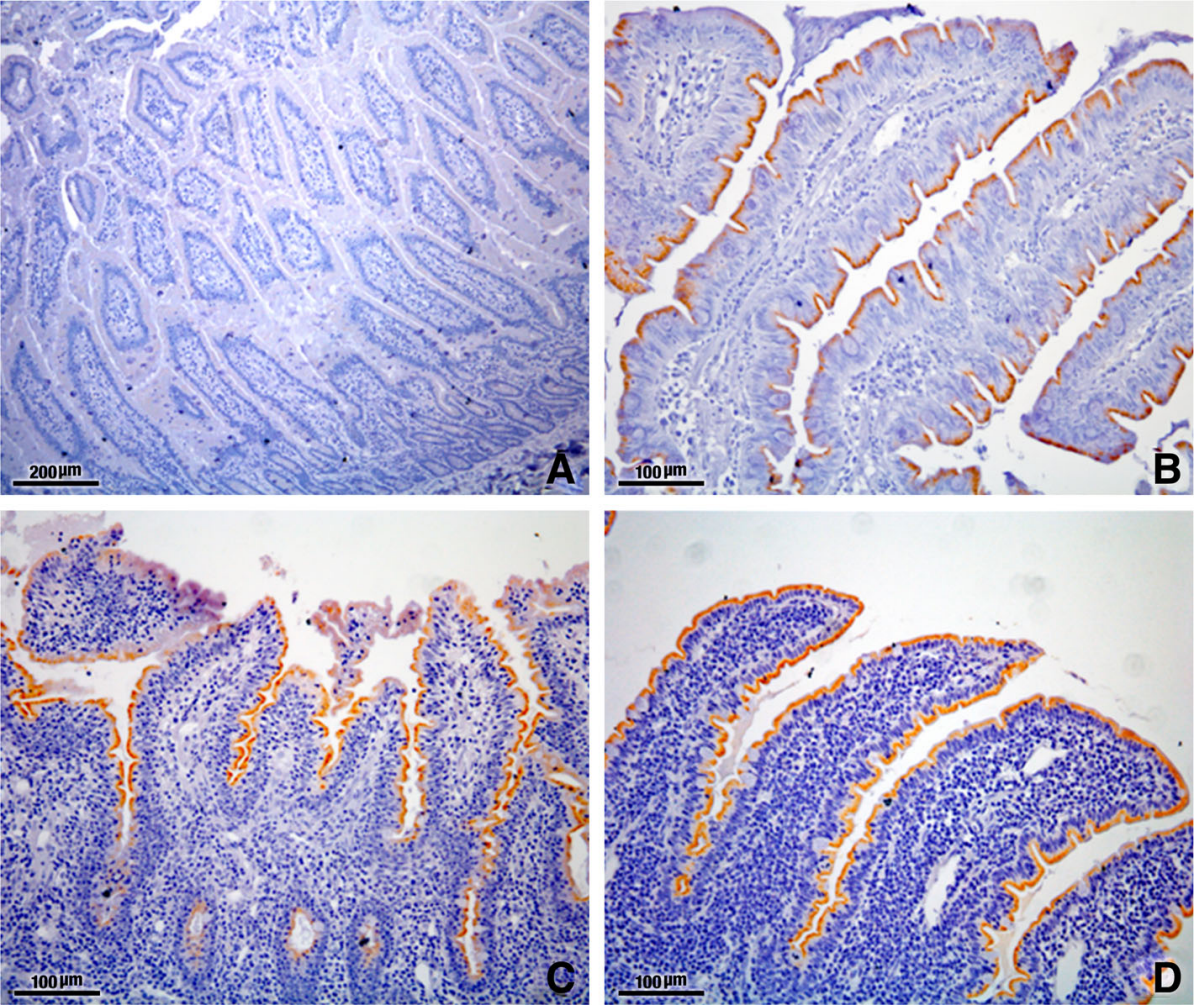
**a** Absence of COX-2 immunolabelling in the apical membrane of the epithelium from duodenum of a sick cat from the control group (score 0); scale bar, 200 μm. **b** Moderate epithelial COX-2 immunoexpression of the apical membrane of enterocytes from the duodenum of healthy control cats (score 2); scale bar, 100 μm. **c** Marked epithelial COX-2 labelling of the apical membrane of enterocytes from the jejunum of severe lymphoplasmacytic enteritis (score 3); scale bar, 100 μm. **d** Marked epithelial COX-2 labelling of the apical membrane of enterocytes from the jejunum of severe lymphoplasmacytic enteritis (score 3); scale bar, 100 μm

COX-2 epithelial immunoexpression was observed in all studied cats, except 3 SC that belong to the control group. Regarding the intensity of expression, 82% of cats with IBD (9 out of 11) and 67% with LGAL (6 out of 9 cats) presented a marked intensity; remaining cats presented a moderate intensity. No significant difference was detected between these groups (*p* = 1.000, Table 1). Sixty-three per cent of cats from the control group showed a moderate epithelial COX-2 intensity, but the other ones did not present staining as mentioned above. Furthermore, control group presented lower intensity in comparison with the IBD (*p* = 0.001) and LGAL group (*p* = 0.008, Table 1 and Fig. 2). Regarding the percentage of cells, all cats from the IBD, 67% (6 out of 9) from the LGAL and 63% (5 out of 8) from the control group showed immunolabelling in more than 60% of the enterocytes, and no statistically significant difference was observed concerning to staining grade (*p* = 0.081, Table 1). COX-2 immunoexpressions are presented in Fig. 3a, b, c and d.

**Fig. 4:**
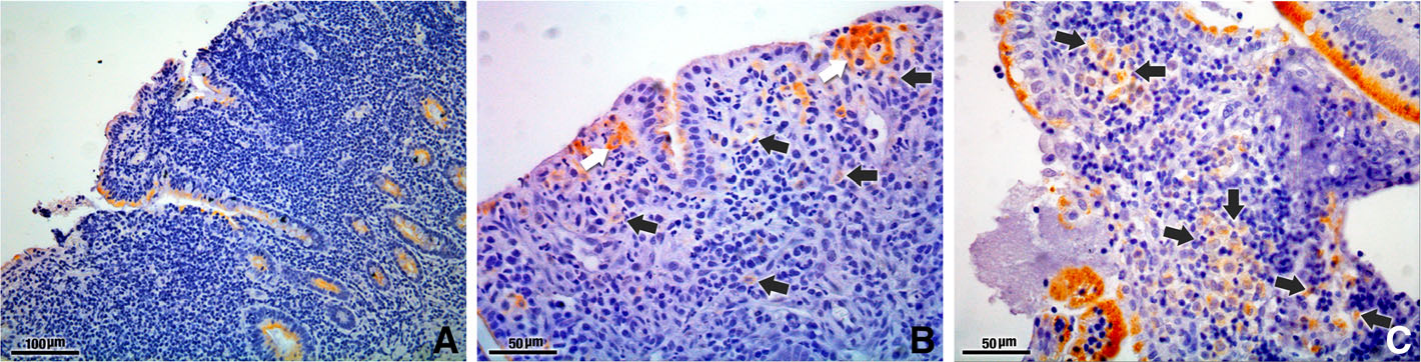
**a** No expression of COX-2 at the neoplastic lymphocytes at the lamina propria of a low grade alimentary lymphoma (intensity 0); scale bar, 100 μm. **b** Moderate COX-2 expression of a few neoplastic cells at the lamina propria (black arrows) and enterocytes with marked reactivity (white arrows) from a cat with low grade alimentary lymphoma (intensity 2); scale bar, 50 μm. **c** Marked expression of COX-2 at some neoplastic lymphocytes at the lamina propria (black arrows) in a cat with low grade alimentary lymphoma (intensity 3); scale bar, 100 μm

COX-2 expression at the LP was absent in all cats from the control and IBD group (Table 1). In the LGAL group, 2 cats presented moderate intensity and 1 cat a marked intensity immunolabelling of neoplastic, however the immunoreactivity was presented in less than 10% of cells (Table 1 and Fig. 4a, b and c). Regardless, no statistical significant differences were observed according to intensity, staining grade and final score of COX-2 expression at the LP between the three groups (*p* > 0.05, Table 1).

Statistically significant lineal correlations were not observed between epithelial or LP COX-2 expression and FCEAI and histological alterations (*p* > 0.05; Spearman’s ρ < 0.354).

## Discussion

The population of animals used in the present study confirmed previous findings showing that IBD affects younger cats compared to AL, although overlap was present. Male cats were overrepresented in LGAL group as well as DSH cats in both studied groups in agreement to previous reports [1–6, 16, 31].

Lymphoplasmacytic inflammation has been the most common inflammatory pattern defined in cats with IBD and was localized most frequently in duodenum [6, 15, 36]. Duodenum is the most common GI segment evaluated, but it is unlikely that IBD is restricted to this segment. This location is probably overrepresented due to limitations of endoscopy to obtain samples from lower small intestine segments. Furthermore, FTBs are likely more obtained from the duodenum as well than the jejunum and ileum like the present study. According to previous reports, T cell LGAL was more frequently localized in the jejunum [5, 6, 11, 16, 37].

In contrast to our findings, a study in cats found correlation between the WSAVA template and the FCEAI [2], however no correlation was observed in studies performed in dogs [1, 29, 38]. These discrepancies might be due to the FCEAI was calculated retrospectively in most of the cats. Furthermore, pancreatitis and hypocobalaminaemia that could worsen clinical signs, was not completely ruled out. Also, in the present study we used FTB from different intestinal segments that have been evaluated by a single pathologist, which might have influenced WSAVA scores.

Maunder and colleagues [31] observed severe duodenal morphological changes applying the MWSAVA scoring in LGAL and herein moderate changes were found. Nevertheless, a significant difference was observed between LGAL and IBD group in the present study regarding MWSAVA scoring. Further studies are needed to determinate whether this histological scoring might help to differentiate between CE.

To our knowledge, this is the first report regarding COX-2 expression in the intestinal epithelium and LP of cats with IBD and LGAL. COX-2 is classically considered an inducible enzyme, but it is also considered a constitutive enzyme expressed in the GI tract [39]. Moreover, COX-2 products might be involved in maintaining the integrity of intestinal mucosa [39]. Differences between species have been described about epithelial COX-2 expression along the GI tract in normal individuals. COX-2 is expressed in the ileocoecal junction and colon in rodents, in all the GI tract in dogs and in the stomach and colon in humans [40–47]. In the present study, cats of the control group presented epithelial COX-2 expression in duodenum, jejunum and ileum (data not shown). Therefore, this supports the need of more studies to clarify COX-2 expression and role in normal individuals.

Regarding immunoreactivity in healthy feline GI tract, only one study described COX-2 immunoexpression in basal granulated cells of the epithelium using a polyclonal antiprostaglandin H synthetase-2 (COX-2) human C terminus antibody [48]. Some differences may be found depending on the antibody used, in our study, immunolabelling was found in the cytoplasm of the enterocytes in 5 cats of the control group (3 HCC and 2 SC). The discordance on inmunoexpression may be explained by the different anti-reagent used, or different affinity of the antibody, however the antibody used herein was previously used in cats [33–35, 45, 46, 49–51]. The presence of COX-2 positive and negative enterocytes in SC of the control group might be explained by the degree of epithelial autolysis in the samples. However, SC were necropsied within 1 h. Even though epithelial autolysis was not observed in the histopathology, molecular autolysis cannot be totally ruled out that could influence on the COX-2 expression. Another possible explanation is the individual variability, it has been demonstrated that only 50 to 80% of healthy humans presents COX-2 expression in colon and stomach [45–47, 52]. Further studies with larger number of cats are needed to obtain conclusions about normal COX-2 expression in the GI tract.

Epithelial intensity immunoexpression in IBD and LGAL groups was significantly higher in comparison with control group though no statistical difference was found between the group of cats with IBD and LGAL. Higher epithelial COX-2 immunolabelling has been reported in humans with gastritis induced by *Helicobacter pylori*, ulcerative colitis or Crohn’s disease compared to normal epithelium. These observations agree with the present study [45–47, 49, 52]. The increased COX-2 expression may be due to GI epithelial ulceration, however in our study only 2 cats with LGAL presented epithelial ulceration (data not shown) [45, 49]. Furthermore, it has been described that COX-2 expression increases after feeding in feline duodenum, but this is unlikely since the cats used in the present study were fasted for anaesthetic procedure or were anorectics [48]. Increased mucosal levels of prostaglandin E_2_ in humans and interleukin-1β in dogs with IBD and food responsive diarrhoea have been linked to an increased COX-2 immunoexpression or upregulation [44, 49]. Based on these studies, it has been suggested that cytokines and prostaglandins induced by an inflammatory response increase COX-2 in the intestinal mucosa as a protective mechanism [44, 49]. Regarding LP, no expression was found in any cat from control or IBD groups. At the same time, the normal feline lymph node did not presented COX-2 expression (data not shown), as previously described in dogs [53]. In humans with IBD, macrophages and polymorphs are stained by COX-2 at the LP [46, 47, 49, 52, 54]. However, those inflammatory cells are not present in feline IBD, and probably for this reason immunolabelling was not found in our cases. Association between COX-2 upregulation and development of lymphoma, as occurs in some tumours, remains unknown but COX-2 overexpression is associated with cell proliferation and angiogenesis [50, 51, 55, 56]. In this study, only 3 cats with LGAL presented COX-2 expression in lymphoid tumour cells. Conversely, Beam and colleagues [26] did not find COX-2 immunoexpression in 6 cats with AL. This disagreement may be due to a different immunohistochemical technique. A recent report stated that 15% of canine lymphoma presented COX-2 overexpression which agrees with the present findings [57]. However, other studies in canine lymphoma did not find COX-2 immunoreactivity [53, 56]. Furthermore, studies in humans revealed that most of non-Hodgkin’s lymphoma (> 50% of cases) had COX-2 expression by tumour cells [58–60]. Thus, COX-2 upregulation in lymphomas has been associated with the aggressiveness, relapsed, worst response to therapy and less overall survival [58–61]. This latter could not be determined in our study because not all cats had available follow-up. Prospective studies are needed in cats with different lymphoma phenotypes and anatomical locations to further understand the role of COX-2 in feline AL. No correlations were observed between FCEAI, histological alterations, IBD and LGAL with COX-2 expression. Similar results have been obtained in canine IBD, and human lymphoma and IBD [44, 46, 58–60].

This study presented some limitations, most of the cases were recruited retrospectively and FCEAI was calculated by record data or owner interview by phone calls, therefore subjectivity may be an uncontrolled variable. Although intraobserver variation among histopathologic evaluations of intestinal tissues were not present due to one pathologist evaluated all biopsies, an intraobserver variation could have existed [62]. Cases of triaditis were not included, however feline specific pancreatic lipase was not available in all cases and pancreatitis was ruled out by ultrasound. Moreover, all histopathological diagnosis was made prior to the availability of polymerase chain reaction for antigen receptor rearrangements; thereby a misdiagnosis could have occurred. However, FTB was available in almost all cats and immunohistochemistry was performed by increasing the sensitivity and specificity [16].

## Conclusions

Increased COX-2 intensity at the epithelial cells observed in cats with IBD and LGAL may be secondary to the inflammatory response or a protective function in the intestinal reparation. COX-2 expression at the LP was presented in only 33% of LGAL cats, thus further investigation of COX-2 expression in feline AL are needed to clarify its importance in prognostic and response to therapy.

## Abbreviations

AL: Alimentary lymphoma
ALP: Alkaline phosphatise
ALT: Alanine aminotransferase
CEs: Chronic enteropathies
COX-2: Cyclooxygenase 2
FCEAI: Feline chronic enteropathy activity index
FTB: Full thickness biopsies
GC: Glucocorticoids
GI: Gastrointestinal
HCC: Healthy control indoor female cats
IBD: Inflammatory bowel disease
LGAL: Low grade alimentary lymphoma
LP: Lamina propria
MWSAVA: Modified WSAVA
SC: Sick cats
WSAVA: World small animal veterinary association
